# Measuring and Modeling Water and Carbon Dioxide Adsorption on Amine Functionalized Alumina under Direct Air Capture Conditions

**DOI:** 10.1021/acs.iecr.4c04581

**Published:** 2025-03-19

**Authors:** Quirin Grossmann, Paola A. Saenz-Cavazos, Nicole Ferru, Daryl R. Williams, Marco Mazzotti

**Affiliations:** † Institute of Energy and Process Engineering, ETH Zurich, Sonneggstrasse 3, 8092 Zurich, Switzerland; ‡ Department of Chemical Engineering, Imperial College London, London SW7 2AZ, U.K.

## Abstract

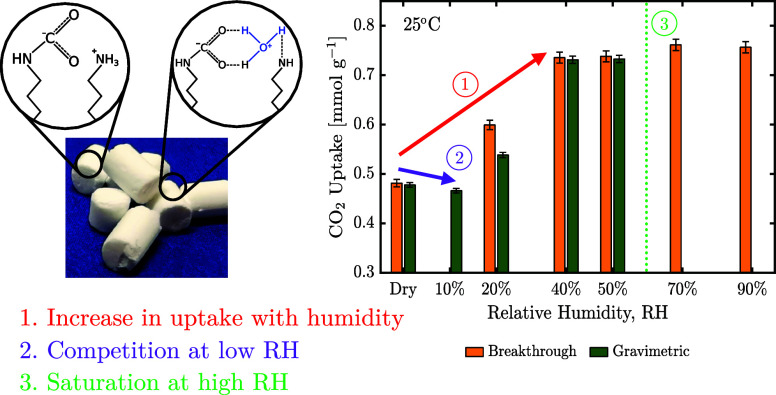

Water vapor is an unavoidable component of ambient air that sorbents designed for atmospheric CO_2_ capture must contend with. Amine functionalized sorbents often exhibit an enhancement of CO_2_ uptake in the presence of moisture through a variety of mechanisms, and in this work, we investigate the coadsorption of water and CO_2_ on amine functionalized alumina. Sorbent performance is examined under varying levels of humidity and temperature using three common measurement techniques: gravimetric, volumetric, and breakthrough methods. Our findings show that water increasingly enhances CO_2_ adsorption up to the monolayer saturation point of water, above which no further enhancement is observed. Competitive adsorption is observed primarily at low relative humidities, and a novel dual-site isotherm model is developed that successfully describes these behaviors. Additionally, this study highlights the unique advantages of each measurement technique for accurately characterizing sorbent performance under direct air capture (DAC) conditions. These insights contribute to the understanding and optimization of amine-based sorbents in DAC applications.

## 1 Introduction

Direct Air Capture (DAC) has emerged as a crucial negative emissions technology in the global effort to mitigate climate change by removing CO_2_ directly from the atmosphere. As CO_2_ concentrations continue to rise, DAC systems provide a pathway to achieving net carbon removal,^1^ complementing emissions reduction strategies.^2^ Various methods of capturing CO_2_ have been developed, which can generally be classified into physical or chemical adsorption/absorption processes. Solid-based adsorbents, particularly those functionalized with amine groups, have attracted considerable interest due to their ability to selectively capture CO_2_ under low partial pressures found in air. However, a key challenge in using solid sorbents lies in their interaction with water vapor, which is omnipresent in the atmosphere and can significantly influence their performance.

Understanding the coadsorption of water and CO_2_ is essential for optimizing the design and application of these materials. Water adsorption can either compete with or enhance CO_2_ capture, depending on the nature of the sorbent and on the operational conditions. Consequently, improving the efficiency and reliability of solid sorbents for DAC requires a deeper understanding of the underlying adsorption mechanisms for both water and CO_2_.

**Figure 1:**
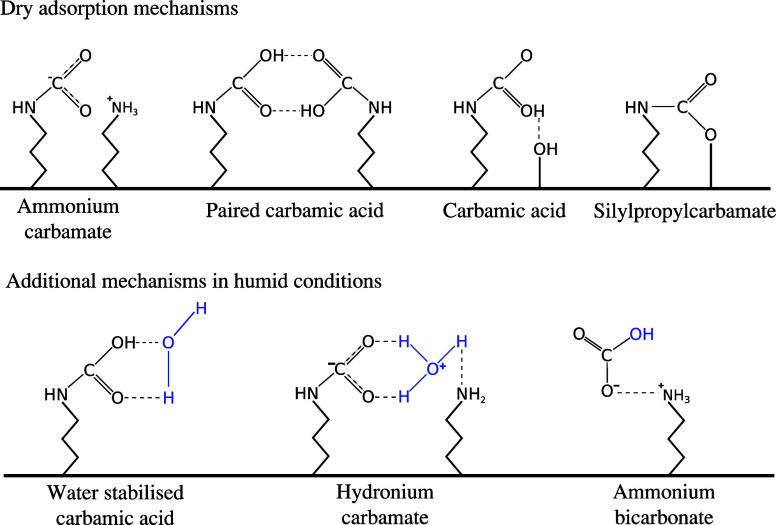
Adsorption mechanisms reported on amine functionalized sorbents under dry and humid conditions.

Water vapor typically enhances CO_2_ uptake of amine functionalized sorbents,^3^ though some cases of competitive adsorption have also been reported,^4−6^ underlining the complexity of the coadsorption mechanisms. On the contrary, in some cases, water adsorption remains unaffected by the presence of CO_2_.^5,7^ Adsorption mechanisms of CO_2_ in dry and in humid conditions reported in literature are illustrated in Figure 1. Under dry conditions, primary and secondary amines chemisorb CO_2_ in the form of ammonium carbamate,^5,6,8−10^ hydrogen bond stabilized carbamic acid,^5,6,8,9,11,12^ and surface bound carbamate.^5,8,11,13^ The nature of the chemisorbed species can depend on the amine coverage, with ammonium carbamate favored at high coverage.^5^ The formation of surface bound carbamate requires the presence of surface OH groups which is typically the case for low amine coverages.^8,11^ Ammonium carbamate is typically the most prevalent species and requires at least two amines to capture one molecule of CO_2_.^5,6,8,14^

Under humid conditions, additional chemisorption pathways are facilitated. While the increase in CO_2_ uptake in the presence of water is sometimes attributed to bicarbonate formation allowing one amine to capture one CO_2_ molecule, this seems unlikely^6,11,15^ as only very small amounts have been observed to form.^5,12^ The catalytic enhancement of carbamate and carbamic acid formation in the presence of water seems the more likely explanation, thereby increasing the amount of these species.^10,11,14−16^ The formation of species with increased amine efficiency, such as water-stabilized carbamic acid,^11^ can further enhance CO_2_ adsorption. The presence of water has further been reported to enhance CO_2_ mass transfer kinetics within the aminopolymers via the formation of diffusive intermediates.^17,18^

The uptake of CO_2_ in the presence of water has been quantified to a varying degree for different amine functionalized sorbents and conditions,^3^ though only few quantify the uptake under DAC conditions. Wurzbacher et al.^19^ observed a steady increase of CO_2_ uptake with increasing relative humidity up to 80% on amine functionalized nanofibrillated cellulose (NFC). This aligns with the observations of Veneman et al.^7^ on the amine functionalized polymeric resin Lewatit VP OC 1065, though in this case only relative humidities up to 60% were measured. The enhancement of CO_2_ uptake has also been reported to plateau beyond a certain relative humidity,^20−23^ or even to decrease at high relative humidities.^6,24^ Furthermore, extensive work, though not necessarily relevant for our study, has been conducted on ionic liquids^25,26^ showing that the presence of water enhances CO_2_ uptake by altering reaction pathways under DAC conditions.

For the quantitative assessment of a DAC process, equilibrium models are required to describe CO_2_ uptake under DAC conditions. Several models have been proposed, which introduce a dependency on the relative humidity through one of these approaches:

1. Modifying the parameters of the dry isotherm^27^;
2. Multiplying the dry isotherm with a function dependent on the relative humidity^28,29^;
3. Combining the dry isotherm with an additional isotherm to define a dual-site isotherm^21,23,29,30^;
4. Using the reaction equations of chemisorption mechanisms, both dry and humid, to calculate the uptake by integration over time.^31^

The models are provided for a limited number of sorbents such as amine functionalized NFC and Lewatit, and often in a limited range of relative humidities, which potentially overlooks effects such as competitive adsorption or plateauing of uptake enhancement.

To further the understanding of coadsorption under DAC conditions, the aim of this study was to investigate the water adsorption properties of an amine functionalized alumina sorbent for DAC applications. In addition, the research provides a comparative analysis of the three most used binary gas adsorption measurement techniques—gravimetric, volumetric, and breakthrough analysis. Table 1 summarizes the advantages, disadvantages and typical sample size of the three different methods. Volumetric gas sorption is widely used due to its versatility across temperatures and pressures, though it can face accuracy issues at low partial pressures and requires complementary methods for multicomponent systems. Gravimetric techniques are advantageous for small sample sizes but require density corrections at high partial pressures and additional analysis for multicomponent systems. Breakthrough or fixed-bed methods stand out with the highest sample size required, though they can provide both equilibrium and kinetic data for multicomponent systems. The analysis presented here provides valuable insights for selecting the appropriate experimental techniques to assess sorbent performance and highlights some of the limitations of the different methods studied.

**Table 1:** Gas Sorption Techniques Comparison Table Including Advantages, Disadvantages, and Typical Sample Size^a^

gas sorption technique	advantages	disadvantages	sample size
volumetric	Most commonly used method, well understood, wide range of temperatures and pressures, amenable to parallelization to enable high throughput.	Pressure gradients between sample and reference can be significant at low partial pressures, possible incremental errors. Requires additional gas analyzing method for multicomponent adsorption.	50 mg^–1^ g
gravimetric	Dynamic mode available that can enhance kinetics, low amount of sample required.	Change in analysis gas density may require additional experimental corrections particularly at higher partial pressures. Requires additional gas analyzing method for multicomponent adsorption.	1 – 50 mg
breakthrough (fixed-bed)	Can provide both equilibria and kinetic information, preferred technique for multicomponent adsorption.	Not suitable for collection of large data sets, not suitable for fine powders.	1 – 10 g

a Reproduced from Saenz-Cavazos et al.,^32^ available under a CC-BY licence. Copyright 2023 Saenz-Cavazos et al.^32^

## 2 Materials and Methods

### 2.1 Materials

The sorbent studied here is a commercial γ-alumina catalyst carrier functionalized with an aminosilane in a wet grafting procedure. The aminosilane consists of two secondary amines and one primary amine, and is grafted onto the cylindrical pellet shaped alumina support using the procedure described in the following.

#### 2.1.1 Reagents

The reagents used for the functionalization were anhydrous toluene (99.8%) and 3-[2-(2-aminoethylamino)-ethylamino] -propyltrimethoxysilane (triamine), purchased from Sigma-Aldrich, and deionized H_2_O (Millipore, Milli-Q Advantage A10). The alumina pellets SA6176 were supplied by Saint-Gobain (France), having a diameter of 3 mm and a length of approximately 3 mm. The triamine and toluene were stored under Ar atmosphere once opened.

#### 2.1.2 Functionalization

The functionalization procedure is based on a wet grafting technique^33^ and is described in detail elsewhere.^34^ In short, 5 g of pellets (dried at 120 °C for 16 h) were added to ca. 120 mL of anhydrous toluene in a 250 mL round-bottom flask onto a stainless steel mesh that separated the pellets from the stir bar, then 75 μL of H_2_O per gram of pellet was added dropwise and left to equilibrate. The temperature was raised to 85 °C and 1 mL triamine per gram of pellet was added dropwise to the solution. After 12 h, the mixture was left to cool to room temperature and the pellets were washed with toluene, ethanol, and diethyl ether.

#### 2.1.3 Material Properties

The properties of the adsorbent used here are characterized in a separate work^34^ using XRD, N_2_ physisorption, Mercury porosimetry, and elemental analysis and are provided in Table 2 as a reference. XRD measurements confirmed the pellets consist of γ-alumina nanoparticles. The reader is referred to the original work for further discussion.^34^

**Table 2:** Sorbent Properties with Values Given per Total Mass of Adsorbent

characteristic	units	value
BJH pore size	nm	7.9
BET surface area	m^2^ g^–1^	99
amine content	mmol_N_ g^–1^	3.6

### 2.2 Gas Sorption

#### 2.2.1 Gravimetric Measurements

Gravimetric sorption measurements were performed using a DVS Resolution (Surface Measurement Systems, UK) using a single pellet with a mass of 32.5 mg. Prior to each measurement, the adsorbent was regenerated at 100 °C for 3 h under vacuum. Given CO_2_ gas was not available in the DVS gas/vapor directory, the Speed of Sound (SoS) sensor was not used to control the gas concentration, instead the instrument mass flow controllers (MFCs) were used. Reservoir A was used for N_2_ and H_2_O, while reservoir B was used for N_2_ and CO_2_, reservoir B was set to operate in an open-loop control mode. A 1.0% CO_2_ in N_2_ cylinder allowed for the generation of low CO_2_ partial pressures to simulate air composition. The maximum relative humidity that could be reached with this setup was 50%. The MFCs were recalibrated using CO_2_ and the following set of equations were derived to calculate the actual CO_2_ concentration accounting for the presence of water vapor:
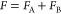
1
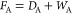
2
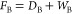
3
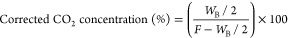
4where *F*, *D*, and *W* are total, dry, and wet flows, while the subscripts indicate flows from reservoir A or B. Both single component and binary gas sorption experiments were conducted with the gravimetric instrument using N_2_ as a carrier gas. Binary isotherms were measured by presaturating the adsorbent with H_2_O and then varying the CO_2_ partial pressure. For the binary gravimetric measurements, we assume that H_2_O adsorption is not affected by CO_2_, and that the CO_2_ adsorption equilibrium on a presaturated material is identical to that of simultaneous H_2_O adsorption.

#### 2.2.2 Volumetric Sorption Measurements

Volumetric sorption measurements were performed on a Microtrac Belsorp Max (Japan) using ca. 100 mg of sorbent per measurement. Prior to each measurement, the adsorbent was regenerated at 100 °C for 3 h at high vacuum. The equilibrium criterion for each measurement point was defined as a pressure change of 0.6% in 20 min. Further restricting the equilibrium criterion to a pressure change of 0.3% in 20 min was found to yield identical results, thus confirming that equilibrium was reached. Deionized H_2_O (Millipore, Milli-Q Advantage A10) was used for the H_2_O sorption measurements, and pure CO_2_ (purity 5.0, Linde) was used for the CO_2_ sorption measurements.

#### 2.2.3 Breakthrough Measurements

All breakthrough measurements were performed on a custom setup described in previous works,^35−37^ which allows for both dry and humid breakthrough experiments. The setup is shown in Figure S1, and has gas bottles of pure N_2_ (purity 5.0, Linde) and CO_2_/N_2_ (400 ppm of CO_2_, purity ± 1% rel., Linde). The inlet flow can be humidified using a Controlled Evaporation Mixing (CEM) system (Bronkhorst, Switzerland) where liquid water drawn from a pressurized water tank is dispersed, mixed with the carrier gas, and vaporized. The entire pipe network is heated with resistive wires to prevent water condensing in the setup. The gas composition at the outlet is analyzed using a capacitive relative humidity sensor (Rotronic, Switzerland) to determine the moisture content, and a flow-through infrared CO_2_ sensor (Vaisala GMP343 0–2000 ppm, Finland).

The adsorbent pellets were crushed to enable the use of a smaller bed, and therefore reduce the amount of sorbent required. Prior to loading, the crushed adsorbent was regenerated at 100 °C and high vacuum for 3 h, then weighed. Typically, an amount of ca. 2 g was loaded into the steel adsorption column of 5 mm in diameter and 200 mm in length. The temperature of the column was controlled using a furnace (Memmert UNE-200, Germany), which allowed for temperature control at least 3 °C above room temperature. Dead volume measurements found that its effect is negligible due to the long time span of the measurements (typically ca. 12 h), and the low concentration of CO_2_ in the gas phase compared to the adsorbed phase.

In a typical measurement, the adsorbent was first regenerated at 100 °C using N_2_ as a purge gas until the outlet CO_2_ concentration *y*_out,CO_2__ reached ca. 5 ppm. The column was then allowed to cool to room temperature and closed, after which the flow was switched to the CO_2_ mix through the bypass, humidified to the desired relative humidity, and then switched back to the column at time *t*_1_. Due to the long tail of the CO_2_ breakthrough, the measurements were stopped when *y*_out,CO_2__ reach at least 95% of the inlet concentration *y*_in,CO_2__. The uptake is then calculated by integrating the mole balance of the column:
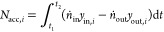
5where *N*_acc,*i*_ is the total amount of species *i* adsorbed and *ṅ* is the total molar flow. The time *t*_2_ was defined by *y*_out,CO_2__ reaching 90% of *y*_in,CO_2__ due to very slow kinetics at long time scales, while for H_2_O it was defined by *y*_out,H_2_O_ = 0.95*y*_in,H_2_O_. The gas inlet flow is controlled by an MFC, which defines *V̇*_g,in_, which is then humidified to *y*_in,H_2_O_. The total molar inlet flow is then defined by
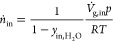
6No flow meter is used at the exit as the concentrations of CO_2_ and H_2_O are very low, therefore the change in concentration due to adsorption has no measurable effect on the outlet flow.^38^ Since N_2_ is assumed to be inert, the flow of N_2_ at the outlet is assumed to be equal to that at the inlet, which gives the following relation for the total outlet molar flow:
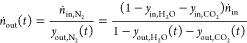
7where *y*_in,CO_2__ is known from the bottle composition, *y*_in,H_2_O_ is set; it is measured with the relative humidity sensor prior to the experiment using the bypass, and then it is assumed to be constant during the whole experiment. The measured uptake *N*_acc,*i*_ is normalized to the mass of regenerated adsorbent to provide *q*_*i*_ in mol kg^–1^.

#### 2.2.4 Sorbent Stability

Amine functionalized sorbents are known to be affected by degradation, the extent of which is influenced by both adsorption and regeneration conditions.^39^ The effect of degradation on CO_2_ uptake was studied for the sorbent used here to inform its effect on equilibrium measurements. Degradation was found to occur to a varying extent depending on the adsorption and regeneration conditions, though could not be entirely avoided. The observations compare to those made in literature,^31,40^ and the sorbent was replaced in regular intervals to minimize its effect on equilibrium measurements. A systematic study of degradation over a significant number of cycles is vital to assess the suitability of a sorbent, though is beyond the scope of this study.

#### 2.2.5 Summary of Equilibrium Criteria

In Table 3 we provide a summary of the equilibrium criteria used for each measurement technique in this study. To enable a comparison, we calculate the equilibrium criteria in mmol min^–1^ for both gravimetric and volumetric methods, essentially describing the change in CO_2_ loading at the defined equilibrium criterion of the corresponding measurement technique.

**Table 3:** Equilibrium Criteria of the Different Measurement Techniques^a^

method	equilibrium criterion		sorbent mass
	(−)	(mmol min^–1^)	(g)
gravimetric	0.0005% Δ*m* per min	4 × 10^–6^	0.035
volumetric	0.6% Δ*p* in 20 min	1 × 10^–7^ (40 Pa, 298 K)	0.1
breakthrough	*y*_out,CO_2__ = 0.9*y*_in,CO_2__		2.0

a Δ*m* refers to the change in mass, and Δ*p* refers to the change in pressure.

### 2.3 Equilibrium Modeling

Several isotherm models are used in this work to describe the measured sorption equilibrium of both CO_2_ and H_2_O on the adsorbent, and are described and discussed in this section. The isotherm parameters are estimated using the Matlab function lsqcurvefit, which further provides the residuals and the Jacobian that are used to determine the 95% confidence intervals (CI) using the Matlab function nlparci.

#### 2.3.1 Sorption Hysteresis

Both CO_2_ and H_2_O isotherms exhibited sorption hysteresis, as shown in Figures S2 and S3 where the observed effects are not due to incomplete equilibrium. This is typically observed for condensable gases, such as water vapor, that experience capillary condensation in small pores.^41^ While CO_2_ adsorption does not conform to this behavior, similar hysteresis has been reported on other amine functionalized sorbents.^29^ This is attributed to the strong chemical bonds binding the CO_2_ requiring heat for their reversal, as a decrease in partial pressure fails to provide enough driving force for desorption. Accordingly, hysteresis is pronounced at low temperatures and effectively disappears at 100 °C. Similar sorption hysteresis is expected for both species for binary sorption, as similar species are formed, though no binary desorption isotherms were measured.

In a typical DAC process, the adsorption step is performed at ambient temperatures and CO_2_ uptake follows the adsorption branch of the isotherm. Desorption is performed at temperatures around 100 °C^42,43^ where negligible hysteresis is observed. Hence, using the adsorption branch to model both adsorption and desorption likely suffices in DAC applications. Accordingly, this work focuses on the modeling of adsorption and neglects any hysteresis effects.

#### 2.3.2 Single Component CO_2_ Adsorption

The temperature dependent Toth isotherm is found to adequately describe dry CO_2_ sorption on the adsorbent used here. It is an empirical correlation that is typically used for adsorbents with submonolayer adsorption on heterogeneous adsorption sites, and is described using the following relation^44^:
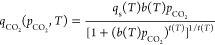
8where *q*_s_ describes the saturation capacity of the adsorbent, *b* is an affinity parameter, and *t* depends on the heterogeneity of the adsorbent. Equation 8 reduces to the Langmuir isotherm when *t* = 1, large deviations of *t* from unity therefore indicate more heterogeneous adsorbents.^44^ The temperature dependence of the Toth isotherm is described through the following relationships:
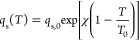
9
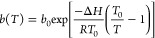
10
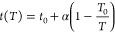
11where Δ*H* is the isosteric heat of adsorption at zero coverage, and the subscript 0 denotes the value of the parameters at the reference temperature *T*_0_.^44^ The fitting of the temperature dependent isotherm is therefore performed in two steps: parameters *q*_s,0_, *b*_0_, and *t*_0_ are estimated by fitting eq 8 to a single isotherm at a reference temperature *T*_0_, then the parameters describing the temperature dependence, χ, Δ*H*, and α, are estimated using isotherms measured at different temperatures.

#### 2.3.3 Single Component H_2_O Adsorption

The Guggenheim-Anderson-de Boer (GAB) extension of the BET isotherm was used as single component water isotherm, as this can describe the multilayer adsorption of H_2_O generally observed for amine functionalized adsorbents.^31^ The GAB isotherm extends the BET isotherm by assuming that the second to the ninth adsorbed layers have heats of adsorption different than the heat of condensation.^45^ The experiments showed that the effect of temperature is accounted for by using the relative humidity rather than the partial pressure of H_2_O, therefore no other temperature effect is modeled. This results in the following relationship as a function of the relative humidity *x*_H_2_O_(45):
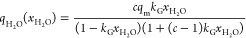
12where *q*_m_ describes the monolayer capacity, *k*_G_ is a constant that accounts for the variation of the heat of adsorption between the second and the ninth adsorbed layer, and *c* is an affinity parameter. Note that if *k*_G_ = 1 eq 12 reduces to the BET equation.^45^

#### 2.3.4 Binary H_2_O and CO_2_ Adsorption

The previously described mechanisms of coadsorption of H_2_O and CO_2_ on amine functionalized adsorbents differ from those on classical physisorbents, where different gases compete for given adsorption sites. Instead, the coadsorption of H_2_O introduces new CO_2_ adsorption mechanisms that lead to changes in adsorption enthalpy and even to the creation of new sites if the amine efficiency is increased, e.g., if CO_2_ is captured in the form of stabilized carbamic acid rather than ammonium carbamate. The coadsorption is therefore mainly collaborative for CO_2_, though some competitive mechanisms cannot be ruled out as the heat of adsorption of some chemisorbed CO_2_ species is significantly lower than that of H_2_O at low coverage. Phenomenologically, the mechanism of coadsorption can be described by a dual-site adsorption isotherm that accounts for some dry adsorption sites (e.g., ammonium carbamate) being replaced by wet adsorption sites (e.g., water-stabilized carbamic acid) with a higher amine efficiency; this has been proposed in several works that describe CO_2_ adsorption using either the Langmuir^21,30^ or the Toth^29^ isotherm. The Langmuir isotherm was unable to describe the dry measurements in this work, the corresponding coadsorption models could therefore not be considered. The dual-site Toth isotherm proposed by Young et al.^29^ will be used here together with a new dual-site Toth isotherm developed in this work. While several other models have been proposed in literature,^23,27,28^ the functional forms fail to describe the phenomena observed here and are therefore not considered. Using the parameters estimated from fitting the dry measurements, the remaining parameters of the humid CO_2_ isotherms are estimated by fitting all the coadsorption data simultaneously, i.e., different relative humidities and different temperatures, with 25 °C as the reference temperature *T*_0_ for the humid isotherms.

##### 2.3.4.1 Weighted Average Dual Site Toth (WADST) Model

The dual site Toth isotherm is essentially an empirical model that differentiates between adsorption sites available under dry conditions and those available under humid conditions. A weighting function is used to describe the contribution of each, and is defined as a function of adsorbed H_2_O. The resulting equation is (Young et al.^29^):
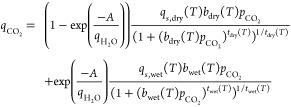
13The coefficients of the dry Toth isotherm are already known from fitting the dry isotherms; the coefficients of the wet Toth isotherm and the critical water loading *A* are estimated by fitting the coadsorption measurements. The temperature dependence of the wet coefficients is given by eqs 9–11, analogous to the dry Toth isotherm, resulting in seven additional parameters to be estimated: *A*, *q*_*s*,wet,0_, *b*_wet,0_, *t*_wet,0_, Δ*H*_wet_, χ_wet_, and α_wet_.

##### 2.3.4.2 Collaborative Adsorption Toth with Site Obstruction (CATSO) Model

Based on the dual site approach, we propose a new empirical isotherm model that includes the main mechanisms influencing the adsorption of CO_2_ in the presence of moisture, namely (1) competitive adsorption for a fraction of the dry adsorption sites, and (2) adsorption on new sites generated upon adsorption of H_2_O. The dual site model based on these two mechanisms is then:
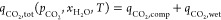
14To account for the first mechanism, we propose a modification of the dry Toth isotherm analogous to the Langmuir multicomponent isotherm^44^:
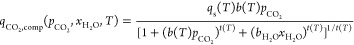
15The affinity parameter of H_2_O, *b*_H_2_O_, is obtained by fitting a Toth isotherm to the H_2_O measurements up to the monolayer capacity, assuming the same heterogeneity parameter *t* obtained from the dry CO_2_ isotherms. Note that it is not a function of temperature due to the use of relative humidity in eq 15. In the second mechanism, we assume that only H_2_O in the first monolayer contributes to the enhancement of CO_2_ adsorption, and that the new adsorption sites can be described by a Toth isotherm. We therefore propose a Toth isotherm with a weighting function correlated to the monolayer coverage of H_2_O:
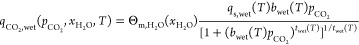
16The “wet” Toth isotherm here describes the sites that are created in the presence of H_2_O, and the temperature dependence of the wet coefficients is again described by eqs 9–11. Θ_m,H_2_O_(*x*_H_2_O_) mechanistically describes the fraction of the first layer occupied by H_2_O, and therefore also the availability of a H_2_O molecule to enhance CO_2_ adsorption. Ideally, this would be a piecewise function consisting of the single-component H_2_O isotherm (eq 12), normalized by the monolayer capacity *q*_m_, up to monolayer saturation at *x*_H_2_O,m_, then remaining constant at one for higher relative humidities. To describe this, we propose the following differentiable function:
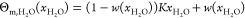
17
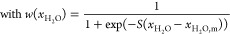
18Here *Kx*_H_2_O,m_ is used to describe the fraction of monolayer saturation and the sigmoidal function *w*(*x*_H_2_O_) describes the transition to the constant value of one at *x*_H_2_O,m_; the parameter *S* describes the sharpness of the transition between the two functions (*S* = 20 is chosen in this work).

The whole model requires the estimation of three parameters for the single-component H_2_O isotherm, namely *b*_H_2_O_, *S*, and *K*, and six parameters for the humid CO_2_ isotherms, namely *q*_*s*,wet,0_, *b*_wet,0_, *t*_wet,0_, Δ*H*_wet_, χ_wet_, and α_wet_.

Both isotherm models require estimating a significant number of additional parameters to describe CO_2_ adsorption in the presence of water, which can increase the uncertainty of their estimation. However, they are essential for capturing the observed mechanisms. In both cases, most additional parameters belong to the Toth isotherm describing newly formed adsorption sites.

## 3 Results and Discussion

### 3.1 Equilibrium Criteria

The measurement of adsorption equilibrium in amine functionalized adsorbents can be challenging due to slow sorption kinetics especially in sorbents impregnated with high molecular weight amine molecules such as PEI,^46,47^ though slow kinetics have also been observed in adsorbents with small, grafted amine molecules.^34,48,49^ The slow kinetics are likely due to diffusion of CO_2_ within the amine layer, which is further inhibited by cross-linking of amine moieties when CO_2_ is adsorbed under dry conditions,^34,49^ and can affect the equilibrium measurements. Care must be taken to ensure enough time is given to reach equilibrium. The equilibrium criteria depend on the physical quantity being measured, and therefore vary for each measurement method, as reported in Table 3.

### 3.2 Single Component Adsorption

#### 3.2.1 CO_2_ Sorption

**Figure 2:**
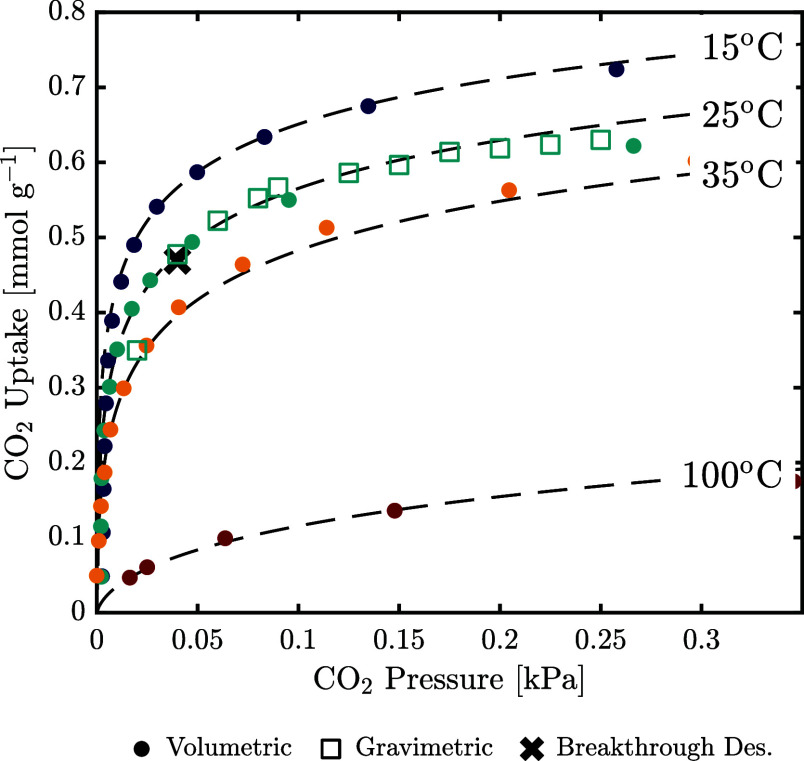
Dry CO_2_ isotherms obtained from volumetric and gravimetric measurements at 25 °C. The uptake measured using a breakthrough at 400 ppm of CO_2_ is provided for comparison. Isotherms were additionally measured at 15, 35, and 100 °C using the volumetric method. The error bars are approximately the size of the symbols and therefore omitted for clarity.

Figure 2 shows the CO_2_ isotherms acquired using volumetric and gravimetric techniques at 25 °C. The CO_2_ uptake at 400 ppm using a breakthrough measurement is also shown for comparison. All measurements are shown to align well, thus confirming the equilibrium criteria chosen for each measurement method.

As the volumetric method demonstrated the fastest equilibration, it was used to measure CO_2_ uptake at different temperatures. The best fit of the Toth isotherm was found using 35 °C as the reference temperature *T*_0_, and the corresponding measurements were used to estimate *q*_s,0_, *b*_0_, and *t*_0_. All other isotherms were then used to estimate the parameters describing the temperature dependence, i.e., Δ*H*, α, and χ. The fitted isotherms are shown in Figure 2 and in Figure S4 for higher pressures, and the values of the estimated coefficients are given in Table 4. Though the isotherm at 35 °C is described well with the estimated coefficients, the confidence interval of the affinity parameter *b*_0_ is very large, indicating a high sensitivity for slight deviations of the other parameters. A physical interpretation of this coefficient is therefore difficult. The parameter describing heterogeneity, *t*_0_, deviates largely from unity, indicating strong heterogeneity in the case of CO_2_ adsorption. The isosteric heat of adsorption at zero coverage, Δ*H*, corresponds well to that observed in similar materials and reported in the literature; it is consistent with the expected combination of adsorption mechanisms, namely, ammonium carbamate (ca. 90 kJ mol^–1^) with a small amount of bound carbamic acid (ca. 40 kJ mol^–1^).^8,9^

**Table 4:** Dry Toth Values

	*T*_0_ (K)	*q*_s,0_ (mmol g^–1^)	*b*_0_ (kPa^–1^)	*t*_0_ (−)	Δ*H* (kJ mol^–1^)	α (−)	χ (−)
value	308	1.17	2.96 × 10^3^	0.25	–77.8	0.19	0.49
95% CI		±0.06	±2.40 × 10^3^	±0.03	±7.6	±0.13	±0.38

#### 3.2.2 H_2_O Sorption

**Figure 3:**
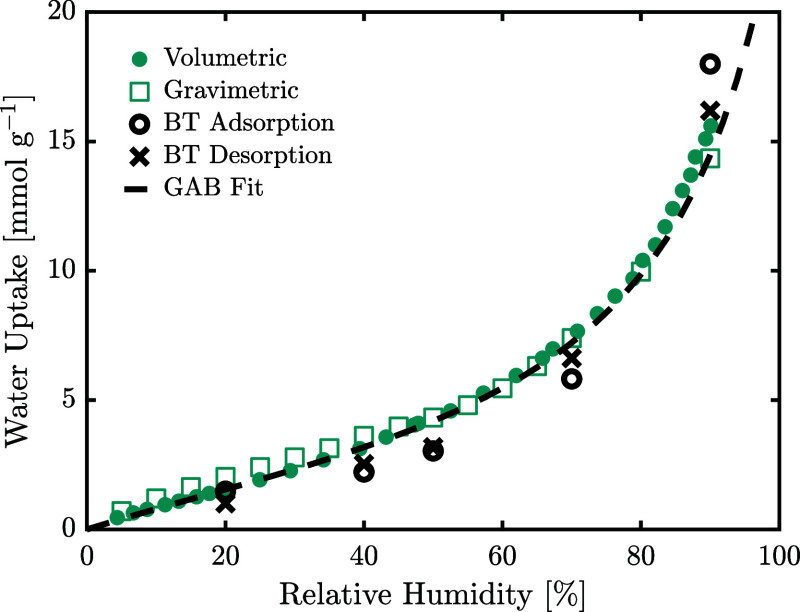
Water isotherms obtained using volumetric, gravimetric, and breakthrough measurements at 25 °C. Isotherms were additionally measured at 15 and 35 °C using the volumetric method and are shown to overlap when described using the relative humidity in Figure S5.

A similar comparative assessment of measurement methods was carried out for H_2_O sorption. Figure 3 shows the single-component H_2_O adsorption isotherms measured using gravimetric, volumetric and breakthrough methods. Both gravimetric and volumetric measurements are shown to align well, as was the case for the CO_2_ isotherms. The breakthrough measurements can be seen to give slightly lower uptakes at all relative humidities, except at 90%. This could be attributed to the slow kinetics and long experimental times associated with breakthrough experiments which can lead to integration errors.^38^ In contrast, breakthrough desorption measurements, being faster, show better alignment with results from other measurement techniques.

The isotherms exhibit a typical IUPAC type III profile describing multilayer adsorption,^50^ which is often seen for H_2_O sorption on amine functionalized sorbents,^31^ and is well described by the GAB isotherm. Isotherms measured at different temperatures are shown in Figure S5 to collapse on each other when plotted against the relative humidity, thus indicating that the temperature effect is accounted for through the variation of the vapor pressure. The estimated parameters are reported in Table 5. The small deviation of *k*_G_ from unity indicates that the adsorbed layers close to the adsorbent surface exhibit a heat of adsorption similar to the heat of condensation. The monolayer saturation capacity *q*_m_ is reached at approximately 43% relative humidity (*x*_H_2_O,m_ = 0.43). Using the Clausius–Clapeyron relation, the average isosteric heat of adsorption for all adsorption layers is ca. 45 kJ mol^–1^, and ca. 59 kJ mol^–1^ at zero coverage. The latter is significantly higher than that of bound carbamic acid (ca. 40 kJ mol^–1^),^8^ thus confirming the possibility of competitive adsorption at low coverage of H_2_O.

**Table 5:** GAB Fit Values

	*q*_m_ (mmol g^–1^)	*c* (−)	*k*_G_ (−)
value	3.51	2.73	0.867
95% CI	±0.12	±0.28	±0.007

### 3.3 Co-Adsorption of CO_2_ and H_2_O

Both the gravimetric and the breakthrough instruments used in this work are suited for coadsorption measurements. Breakthrough measurements are versatile and provide extensive information on the adsorption process as well as on the uptake capacities. However, they are typically slow and therefore unsuited for acquiring a significant amount of uptake data. The gravimetric device used in this work provides an efficient, automated method of acquiring coadsorption data, provided some assumptions can be made. Hence, breakthrough measurements were used to confirm the assumptions required, and the gravimetric method was used to collect the coadsorption data.

#### 3.3.1 H_2_O Sorption

Batch coadsorption measurements of CO_2_ and H_2_O on solid adsorbents are generally performed by presaturating the adsorbent with H_2_O using an inert carrier gas, then stepwise increasing the partial pressure of CO_2_.^5,29^ This inherently assumes that H_2_O adsorption is unaffected by the CO_2_ adsorption, unless the bulk concentration is measured. This has been experimentally proven for amine functionalized cellulose^51^ and a polymeric resin,^7^ though may not hold for high relative humidities.^29^

**Figure 4:**
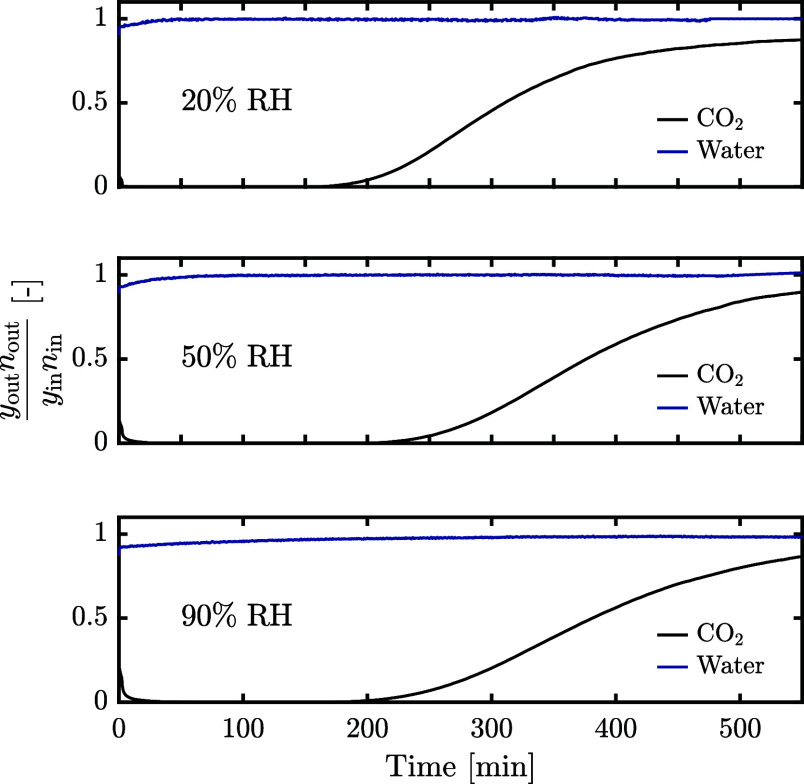
CO_2_ and H_2_O breakthrough profiles on a bed presaturated with H_2_O at different relative humidities. Measurements were performed at 25 °C using 400 ppm of CO_2_ in N_2_.

To test the assumption for the materials used here, breakthrough measurements were designed to mimic the gravimetric measurements: The bed was first saturated with H_2_O in N_2_, after which a CO_2_ /N_2_ mixture was fed. The breakthrough profiles of the CO_2_ adsorption step are shown in Figure 4 at three different relative humidities. A displacement of H_2_O by CO_2_ would be visible as a roll-up, i.e., an increase of the normalized molar outlet flow of H_2_O above one.^38^ This is not observed even at a high relative humidity, indicating that the assumption is valid. It should be noted that, given the long duration of the adsorption step and the precision limits of the RH sensor, small roll-up effects may remain undetected.

A second inherent assumption is that CO_2_ adsorption on a presaturated adsorbent is equal to that of simultaneous CO_2_ and H_2_O adsorption. This is confirmed by comparing the CO_2_ uptake of sequential and simultaneous breakthrough measurements, as shown in Figure S6. In fact, the breakthrough profiles presented in Figure S9a show that water saturates the sorbent bed much quicker than CO_2_, hence the adsorption of CO_2_ essentially occurs on a presaturated bed even for simultaneous adsorption measurements. The protocol typically used in gravimetric measurements therefore is consistent with the breakthrough measurements.

#### 3.3.2 CO_2_ Sorption

**Figure 5:**
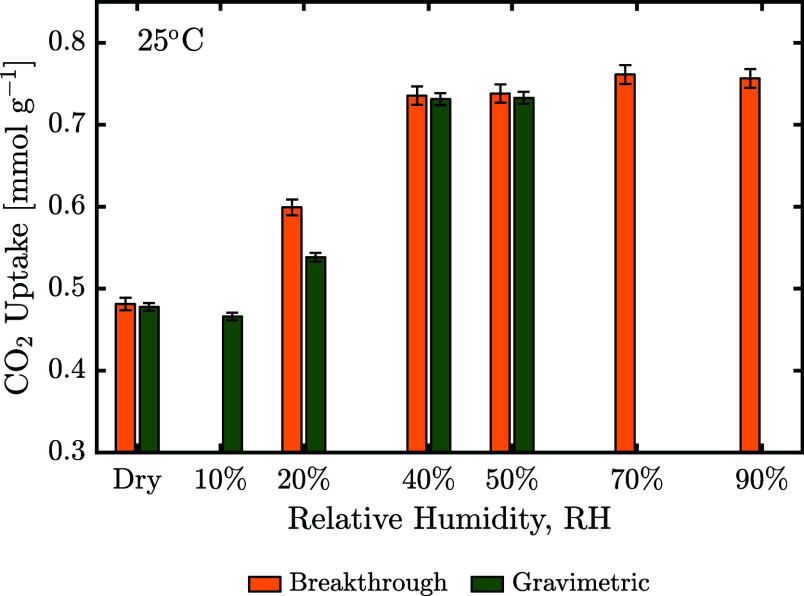
Effect of relative humidity on CO_2_ uptake at 400 ppm of CO_2_ at 25 °C, using both breakthrough and gravimetric measurements. The measurement at 10% relative humidity was performed thrice.

The uptakes of breakthrough measurements at 400 ppm of CO_2_ and different relative humidities are compared to the gravimetric measurements in Figure 5. The CO_2_ uptake at 10% relative humidity (RH) could not be measured with breakthrough techniques due to large humidifier-induced RH fluctuations, and the gravimetric device was limited to 50% RH. The uptakes are shown to be in good agreement under dry conditions, at 40%, and at 50% RH. A deviation between measured uptakes at 20% RH may indicate a small amount of water being displaced through competitive adsorption, though this could not be observed in the breakthrough measurements. In this case, CO_2_ adsorption would be slightly underestimated using the gravimetric method, potentially also explaining the decrease in CO_2_ uptake at 10% RH compared to dry conditions.

However, an initial decrease of CO_2_ capacity at low relative humidity has also been reported elsewhere,^5,52^ and can be attributed to H_2_O blocking access to some adsorption sites. Water may additionally compete with weakly bound CO_2_ species, such as stabilized carbamic acid (see Figure 1).

With the exception of 10% RH, the presence of H_2_O is shown to enhance the capacity of CO_2_ across the whole humidity spectrum. Interestingly, the enhancement reaches a plateau at around 40% relative humidity, approximately the humidity at which the monolayer capacity of H_2_O is reached. Based on the mechanisms shown in Figure 1, water can only enhance adsorption of CO_2_ when it is in the vicinity of the amines, hence multilayer adsorption of water does not lead to a further increase in CO_2_ adsorption. Only breakthrough measurements are shown at high relative humidity due to inherent gravimetric setup limitations.

Both competitive and collaborative adsorption mechanisms are likely present at all relative humidities, with their contrasting effect on CO_2_ adsorption. While competitive adsorption dominates at very low relative humidities, its effect on CO_2_ uptake is compensated and surpassed by collaborative mechanisms at higher relative humidities.

Both competitive adsorption^4,5^ and a plateau of CO_2_ uptake at increasing relative humidities^20−24^ have been reported previously, though often the number of humidity levels explored is too low to observe these effects.^29,40,51,53−55^ Other works do not observe a saturation effect with increasing relative humidity,^7,19,30,31,56^ making it challenging to provide a coadsorption model that describes well all the observations. Table S1 provides a comparison of the material used in this work with those reported in the literature.

### 3.4 Modeling Co-Adsorption

**Figure 6:**
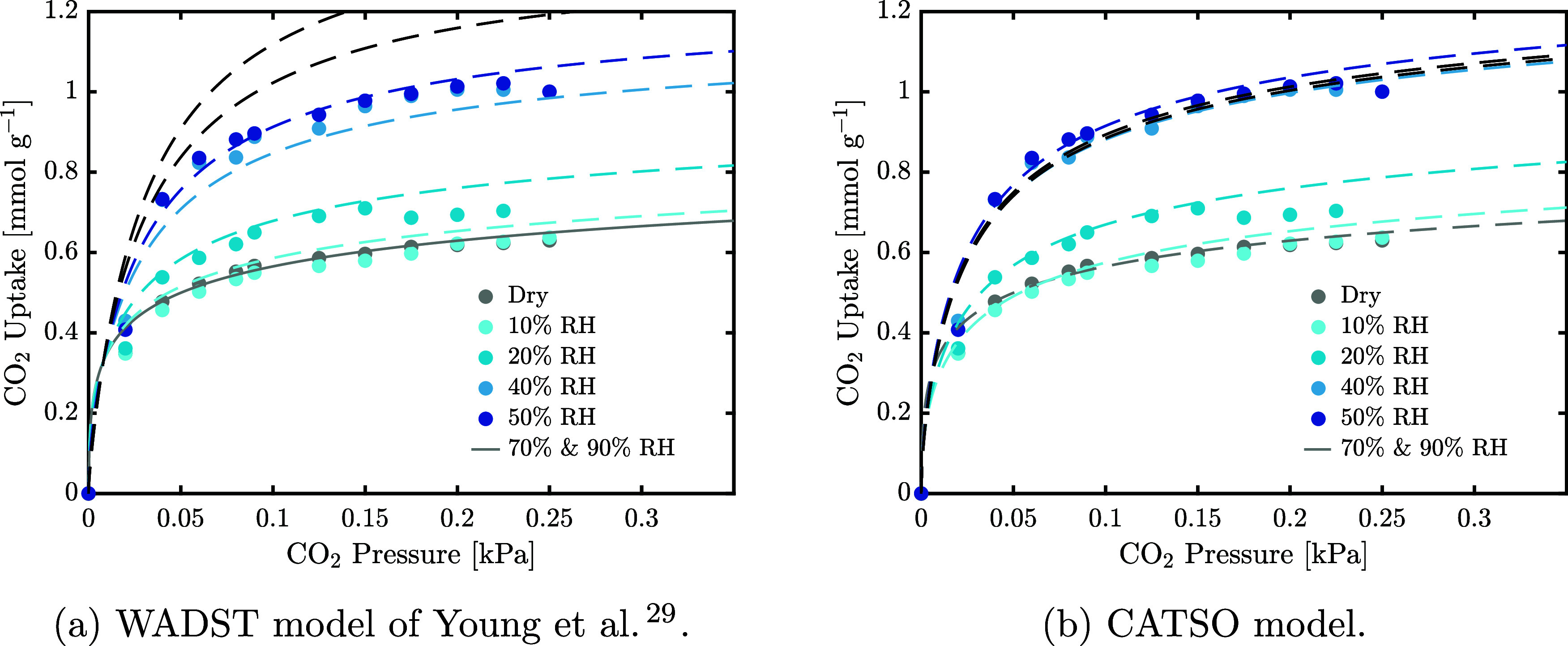
Humid CO_2_ isotherms measured at 25 °C at different relative humidities with fitted models. The black dashed lines are modeled isotherms at 70% and 90% relative humidity. Panel (a) shows the WADST model fit, and (b) the CATSO model fit respectively.

In Figure 6 we show the gravimetric coadsorption data together with the two isotherm models presented previously, fitted to the gravimetric and breakthrough coadsorption measurements. Both models describe the data well for low relative humidities, i.e., up to 50% RH, with the CATSO model being able to describe even the competitive behavior observed in the 10% RH isotherm. The saturation effect above ca. 40% RH is well described by the CATSO model even when extending the model to 70 and 90% (see the black dashed lines in Figure 6b). The WADST model is unable to capture the saturation effect, leading to an overestimation of the CO_2_ capacity at high relative humidities. Sorbents that display such a saturation behavior are therefore better described by the CATSO model.

The estimated parameters of both models are reported in Table 6 and provide qualitative indications of coadsorption effects. Note that parameters *S*, *K*, and *b*_H_2_O_ are estimated using the single-component H_2_O measurements. Due to the form of the WADST model, its wet isotherm describes all adsorption mechanisms active under humid conditions, including those active under dry conditions. Correspondingly, its saturation capacity *q*_s,0,wet_ is larger than that of the dry isotherm to reflect the collaborative nature of coadsorption. In the CATSO model, the wet isotherm describes the *additional* adsorption that occurs in the presence of water through mechanisms shown in the lower section of Figure 1. The sum of *q*_s,0,wet_ and *q*_s,0,dry_ in the CATSO model (see additionally Table 4) is larger than *q*_s,0,wet_ of the WADST isotherm as it compensates for competitive adsorption in addition to describing the enhancement of CO_2_ capacity.

**Table 6:** Estimated Parameter Values of the Fitted Co-Adsorption Isotherms Shown in Figures 6 and 7^a^

		WADST model^29^		CATSO model	
parameter	units	value	95% CI	value	95% CI
*q*_s,0,wet_	mol kg^–1^	1.62	±0.31	0.60	±0.12
*b*_0,wet_	kPa^–1^	31	±17	40	±31
*t*_0,wet_		1.0	–b	1.0	–b
Δ*H*_wet_	kJ mol^–1^	0	–b	0	–b
α_wet_		4.9	±7.7	6.2	±9.3
χ_wet_		2.3	±3	0.2	±3.4
*A*	mol kg^–1^	2.8	±0.5		
*S*				20^c^	–d
*K*				2.28^c^	±0.002
*b*_H_2_O_				0.064^c^	±0.93

a The parameters *S*, *K*, and *b*_H_2_O_ in the CATSO model are estimated using the single-component H_2_O measurements.

b No CI available due to value reaching physical parameter bounds.

c Estimated using the single-component H_2_O isotherm.

d This value is chosen, not estimated, and therefore has no CI.

The affinity parameters *b*_0,wet_ are 2 orders of magnitude smaller than that of the dry isotherm for both models, indicating that the additional species are weakly bound, as would be the case for water-stabilized carbamic acid^57^ (see Figure 1). The value of the heterogeneity parameter *t* is found to be a result of the low partial pressure range of the coadsorption measurements rather than of a physical phenomenon.

#### 3.4.1 Temperature Dependence of Co-Adsorption

**Figure 7:**
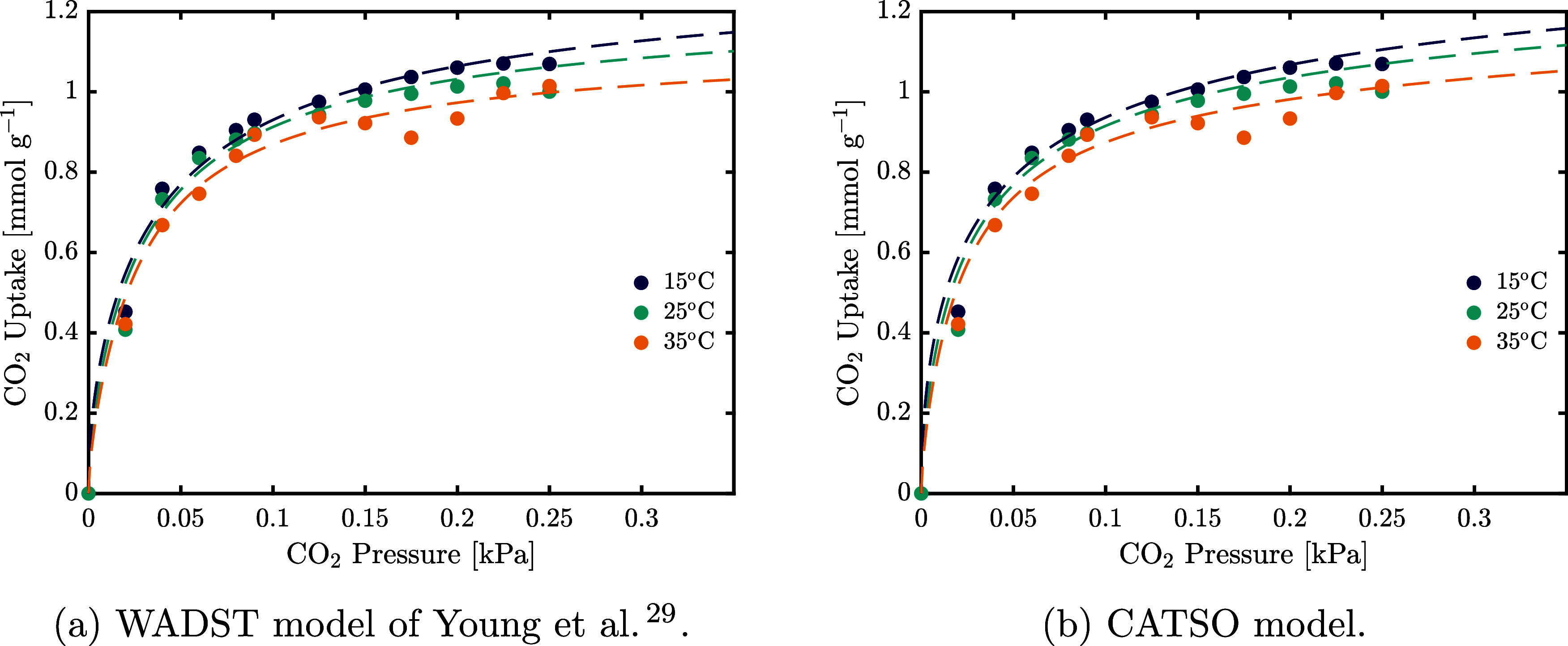
Experimental results of humid CO_2_ adsorption at 50% relative humidity measured at 15, 25, and 35 °C, with the fitted adsorption isotherms. Panel (a) shows the WADST model fit, and (b) the CATSO model fit, respectively.

The temperature dependence of the coadsorption isotherms shown in Figure 7 is weak compared to that of the dry isotherms, as observed elsewhere as well.^19^ The isotherms are measured at the same relative humidity, indicating that the temperature dependence of CO_2_ adsorption under humid conditions is primarily governed by the temperature dependence of the water vapor pressure within the measured temperature range. To maintain broader applicability and validity over larger temperature ranges, the temperature dependence observed is described in the model, using the parameters reported in Table 6. The parameters describing the temperature dependence, i.e., α_wet_ and χ_wet_, have large confidence intervals and the estimated isosteric heat of adsorption Δ*H*_wet_, which is negative, reaches its physical upper bound of zero, further underlining the weak temperature dependence. The models fitted assuming no temperature dependence of the wet adsorption term are shown in Figure S8 for reference and show an acceptable fit in both cases.

## 4 Conclusions

Single and multicomponent adsorption of CO_2_ and water on amine functionalized alumina was investigated using common measurement methods with the goals of (1) understanding and quantifying CO_2_ and water coadsorption under direct air capture conditions and (2) comparing widely used measurement methods. Understanding the effect of humidity on the adsorption potential of amine functionalized sorbents is crucial for optimizing DAC cycles under real conditions. Of course, its effect on desorption also needs to be understood to fully optimize a cycle; this aspect, though very relevant, goes beyond the scope of this work.

The measurements revealed that water enhances CO_2_ uptake, while competitive adsorption remains minor and is observed mainly at low relative humidity levels. Water adsorption is unaffected by the presence of CO_2_. The enhancement in CO_2_ uptake in the presence of water vapor plateaus around the monolayer saturation point of water, with excess relative humidity neither further enhancing nor hindering CO_2_ adsorption. The effect of temperature on CO_2_ uptake under humid conditions is minimal and appears to be governed by the temperature dependence of the water vapor pressure.

A new isotherm model was developed to describe the experimental observations, effectively capturing both competitive and collaborative adsorption mechanisms. Given that coadsorption mechanisms can vary among different sorbents, the choice of isotherm model should be based on experimental observations. Measurements at enough different relative humidities are crucial to capture both competitive and collaborative effects.

Among the measurement methods used, the volumetric device provided useful single component data, but was found unsuitable for measuring multicomponent adsorption. While breakthrough measurements offer detailed insights on adsorption processes, they are time-intensive and require substantial manual labor. With certain assumptions in place, gravimetric measurements can efficiently collect a significant amount of multicomponent adsorption data in an automated manner.

## Supporting Information Available

Additional information on methods and materials, functionalized pellets, spectroscopy measurements, and mass transfer kinetics is given in the Supporting Information (PDF)
