# Supplementary material for: Measuring and Modeling Water and Carbon Dioxide Adsorption on Amine Functionalized Alumina under Direct Air Capture Conditions
Source: Ind Eng Chem Res. 2025 Mar 19;64(13):7165–75. doi: 10.1021/acs.iecr.4c04581 (PMC11969556; doi:10.1021/acs.iecr.4c04581)
Supplement: Supplementary file 1 — ie4c04581_si_001.pdf [file ie4c04581_si_001.pdf]

Supporting Information: Measuring and Modelling Water and Carbon Dioxide Adsorption on Amine Functionalized Alumina under Direct Air Capture Conditions

Quirin Grossmann,[†] Paola A. Saenz-Cavazos,[‡] Nicole Ferru,[†] Daryl R. Williams,[‡] and Marco Mazzotti[*,†]

†*Institute of Energy and Process Engineering, Sonneggstrasse 3, ETH Zurich, 8092 Zurich, Switzerland*

‡*Department of Chemical Engineering, Imperial College London, London SW7 2AZ, UK*

E-mail: marco.mazzotti@ipe.mavt.ethz.ch

Methodology

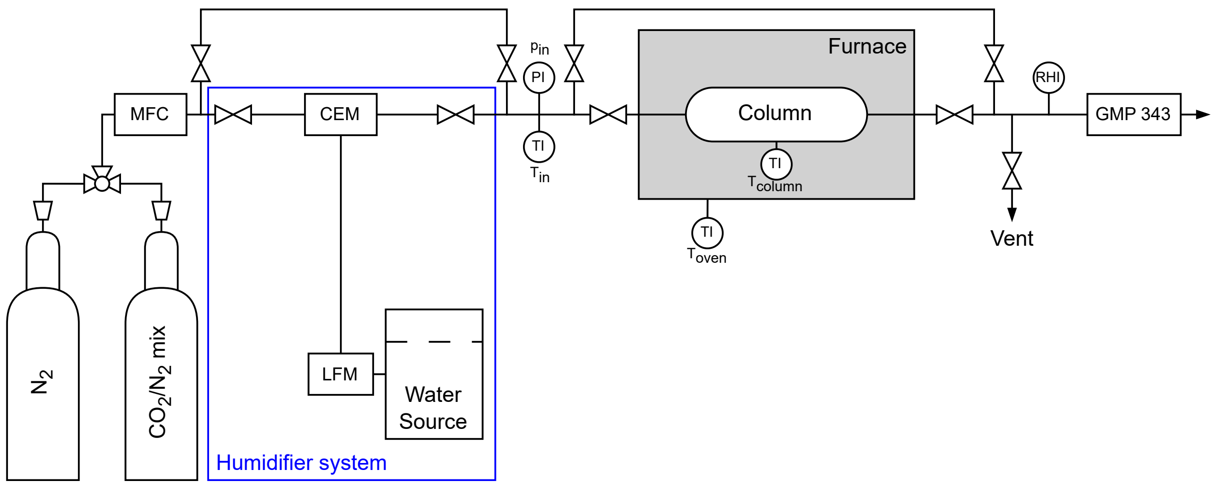

Figure S1: Flow sheet of the breakthrough measurement setup composed of a feeding system including a humidifier, a temperature controlled column, and a gas analysis block that allows to determine the moisture content and CO_2 concentration at the outlet. Abbreviations used: MFC, mass flow controller; CEM, controlled evaporative mixer; LFM, liquid flow meter; TI, temperature indicator (thermocouple); PI, pressure indicator; RHI, relative humidity indicator; GMP 343, CO_2 sensor

Sorption Hysteresis

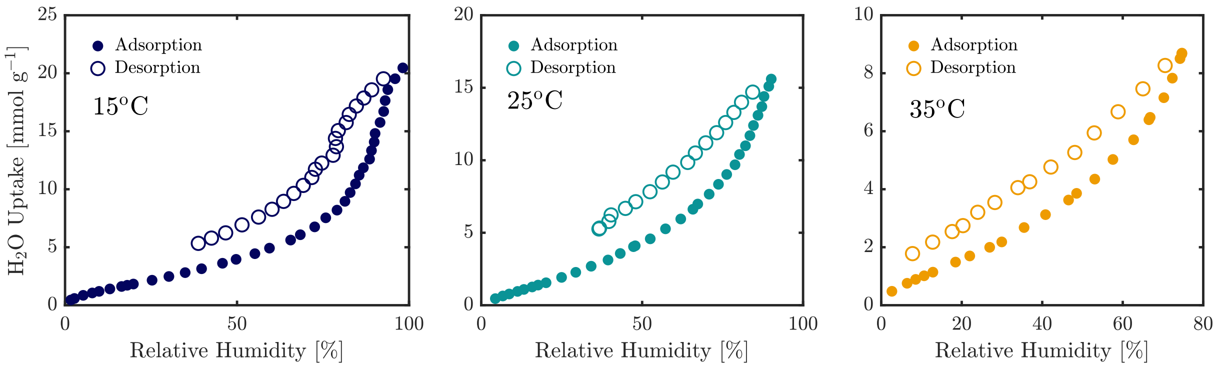

Figure S2: Hysteresis in H_2O isotherms.

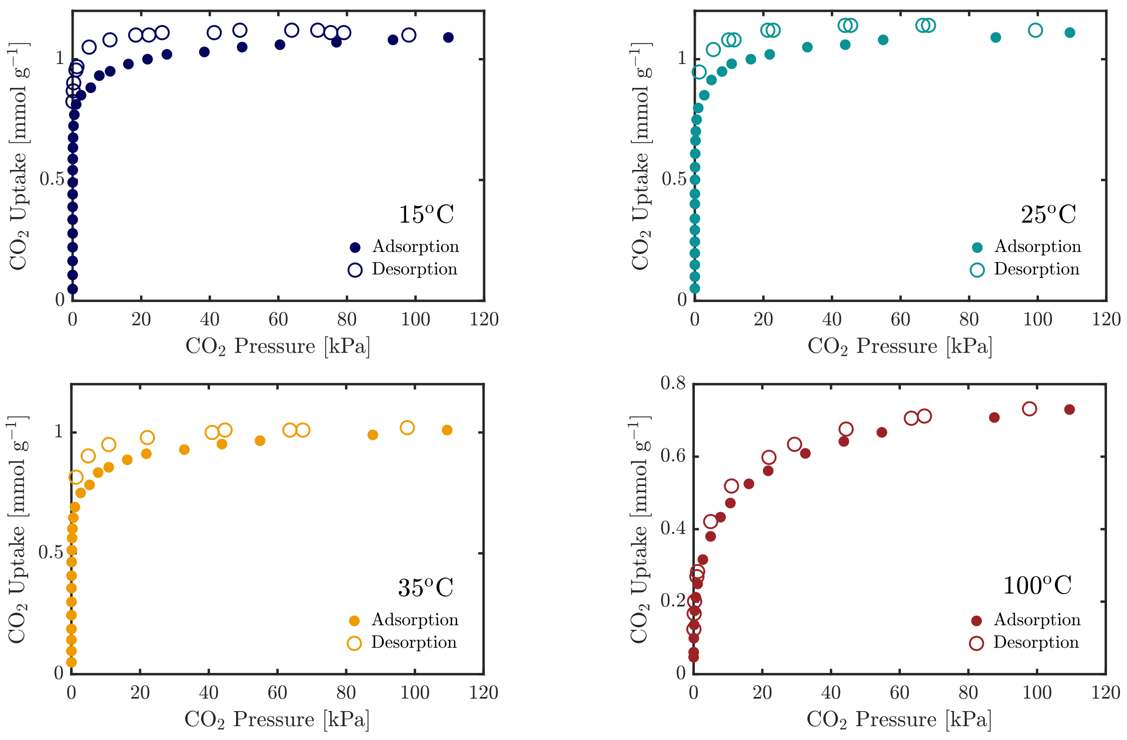

Figure S3: Hysteresis in CO_2 isotherms.

Single Component Adsorption

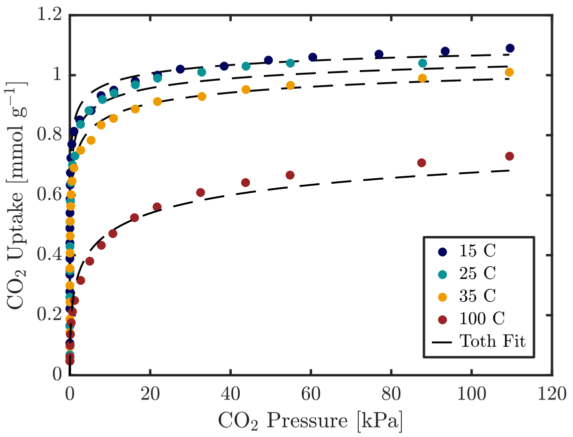

Figure S4: Dry CO_2 isotherms obtained using volumetric at different temperatures up to 100 kPa.

Water isotherms were measured at three different temperatures using the volumetric method and are shown in Figure S5. Plotted against the relative humidity, they collapse onto each other, showing that the temperature dependence can be described by the change in vapour pessure.

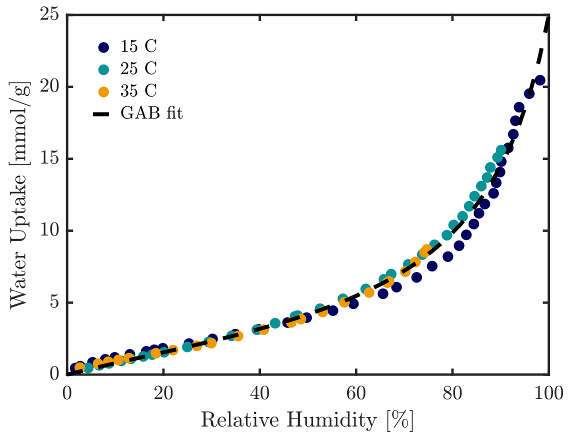

Figure S5: GAB isotherm fitted to water adsorption measurements using the volumetric method at three different temperatures.

Co-Adsorption of CO_2 and H_2O

Presaturation Breakthrough Measurements

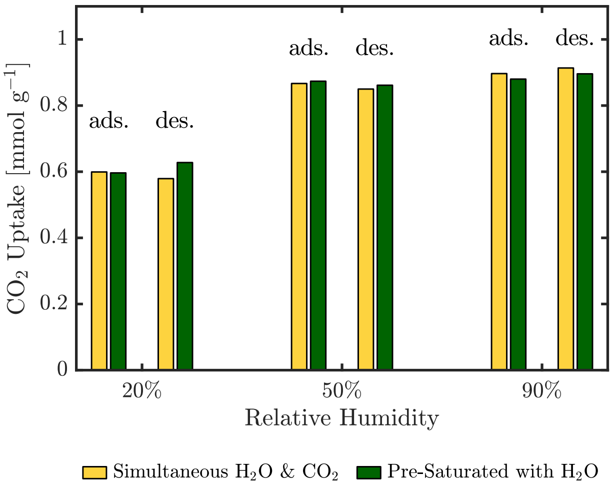

Figure S6: Comparison of the CO_2 uptake with simultaneous and sequential H_2O and CO_2 adsorption at 25°C using breakthrough measurements. Sequential adsorption involves pre-saturating the bed with water followed by CO_2 at 400 ppm at the same relative humidity. Both adsorption and desorption measurements are shown for comparison.

CO_2 Adsorption

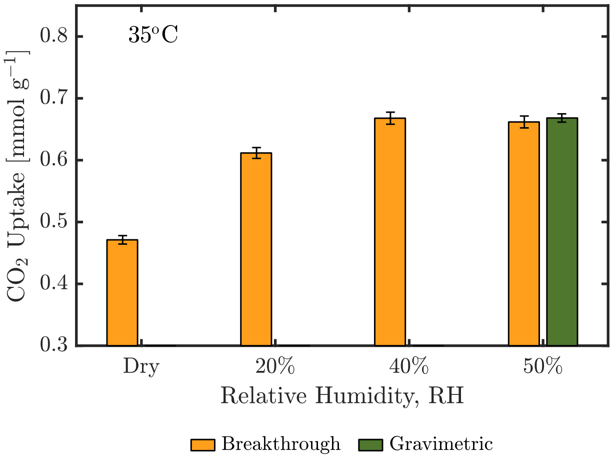

Figure S7: Effect of relative humidity on CO_2 uptake at 400 ppm CO_2 at 35°C, using both breakthrough and gravimetric measurements.

Temperature Independent Model

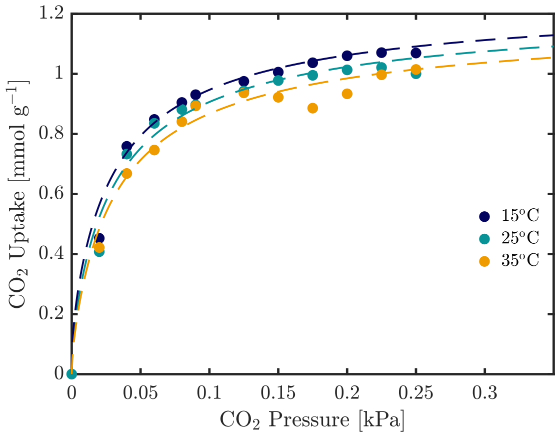

(a) WADST model of Young et al. [1].

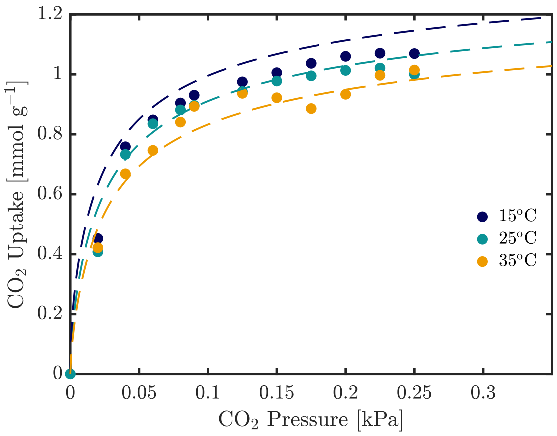

(b) CATSO model.

Figure S8: Experimental results of humid CO_2 adsorption at 50% relative humidity measured at 15°C, 25°C, and 35°C, with the fitted adsorption isotherms. The term describing wet adsorption is assumed to be temperature independent. Panel (a) shows the WADST model fit and (b) the CATSO model fit respectively.

In Figure 8 the isotherms are shown assuming that the term describing the wet adsorption of CO_2 is independent of temperature. This essentially reduces the number of parameters required by three for each model.

Breakthrough Profiles

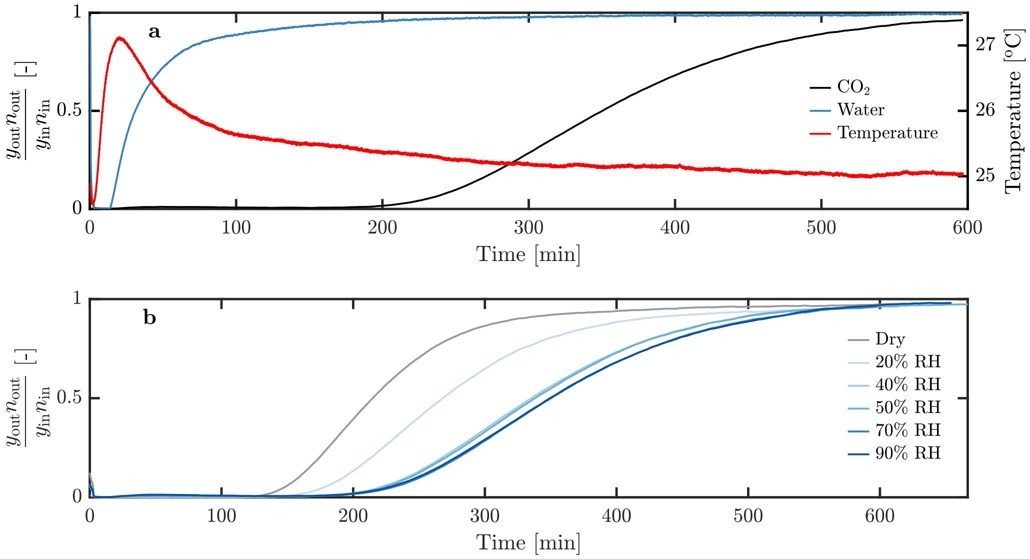

Figure S9: H_2O and CO_2 breakthrough profiles performed at 25°C. The top figure (a) shows both H_2O and CO_2 breakthrough profiles at 70% relative humidity, together with the temperature measured at the center of the column. The bottom figure (b) shows the CO_2 breakthrough profiles at different relative humidities.

Breakthrough profiles contain much more information than equilibrium capacity, and in this section we discuss the various aspects observed in those measurements. Figure 9a shows exemplary breakthrough profiles using 400 ppm CO_2 in N_2 at 25°C and 70% relative humidity. The water breakthrough is shown to occur significantly earlier than that of the CO_2 despite its larger adsorption capacity, owing to the higher concentration of water in the feed. Hence, CO_2 adsorption essentially occurs on a pre-saturated bed similar to the measurement protocol performed in gravimetric measurements. Figure 9b shows the breakthrough profiles at different relative humidities between 0 to 90% relative humidity. The increase in CO_2 uptake with increasing relative humidity is evident from the increase in breakthrough time, which also shows the saturation effect above 40% – 50%.

The slope of the breakthrough profiles give an indication of the mass transfer kinetics.[2] The profiles are shown to flatten at higher relative humidities, indicating slower mass transfer kinetics in the presence of water. This is likely due to the dependence of pore mass transfer

kinetics on equilibrium uptakes.[3,4] Other explanations, such as the formation of a water barrier,[5] seem unlikely as the kinetics stay very similar in a large range of relative humidities where the water uptake changes significantly.

Many dry CO_2 mass transfer studies observe and account for two mass transfer regimes in amine functionalized sorbents[4,6,7] - a fast mass transfer regime often assumed to correspond to pore diffusion, and a slow mass transfer regime often assumed to correspond to diffusion or reactions in the amine layer. Cross-linking of amines due to CO_2 adsorption is assumed to further decrease diffusion in the amine layer, and causes long tails in the breakthrough profiles as observed for dry and 20% relative humidity. This behaviour is less pronounced at higher relative humidities, indicating faster diffusion in the amine layer in the presence of humidity. This may be due to the water promoting diffusion via diffusive intermediate species,[8,9] as well as decreasing cross-linking by promoting additional adsorption mechanisms.

Comparison with Other Reported Materials

Table S1: Literature Comparison

Ref.	Material	Temperature [K]	CO_2 Conc.	H_2O Conc.	CO_2 Uptake [mmol g^{-1}]	Measurement type
This work	Alumina, TRI	298	400 ppm	dry	0.48	TGA & Break-through
				10% RH	0.47	
				20% RH	0.6	
				40% RH	0.74	
				50% RH	0.74	
				70% RH	0.76	
				90% RH	0.76	
		308		dry	0.47	
				20% RH	0.61	
				40% RH	0.67	
				50% RH	0.66	

Avellaneda et al.[10]	SBA-15, APTES	303	10 vol.%	dry	0.4	Breakthrough
				10% RH	0.47	
				20% RH	0.54	
				50% RH	0.70	
Elfving et al.[11]	Amine-functionalized resin	298	400 ppm	2 vol.%	0.89	Breakthrough
		308			0.61	
		323			0.36	
Elfving and Sainio[12]	Amine-functionalized resin	298	380 ppm	2 vol.%	0.87	Breakthrough
Didas et al.[13]	SBA-15, APTMS	303	0.0004 bar	dry	0.6[a]	Volumetric
				2 mmol g^{-1}	0.52[a]	
Belmabkhout et al.[14]	PE-MCM-41, TRI	298	400 ppm	dry	0.90	Breakthrough
				27% RH	1.19	
				64% RH	1.40	
Gebald et al.[15]	NFC, APDES	296	0.04 kPa	dry	1.11	Breakthrough
				2.55 kPa	2.13	
		323		dry	0.11	
				2.55 kPa	1.42	

Wurzbacher et al.[16]	NFC, APDES	283	0.04 kPa	20% RH	0.36	Breakthrough
				80% RH	0.63	
		293		20% RH	0.39	
				80% RH	0.65	
		303		20% RH	0.32	
				60% RH	0.50	
Hahn et al.[5]	SBA-15, TEPA	323	10 vol.%	dry	1.1	Breakthrough
				5 vol.%	1.6	
				10 vol.%	1.9	
				12 vol.%	2.2	
He et al.[17]	RFAS, APTES	303	1 vol.%	dry	1.48[a]	Breakthrough
				1.0 vol.%	1.90[a]	
Holmes et al.[18]	M_2(dobpdc), Diamine	313	4.5 vol.%	dry	2.0[a]	Breakthrough
				10% RH	2.3[a]	
				20% RH	4.4[a]	
				30% RH	4.5[a]	
				50% RH	1.6[a]	
Jung and Lee[19]	fumed silica, EP-PEI	313	2.5 kPa	dry	2.15[a]	Breakthrough
				2.5 kPa	1.6[a]	
				3.75 kPa	1.72[a]	

Liu et al.[20]	KIT-6, TEPA	333	10 vol.%	dry	2.58	Breakthrough
				10% RH	2.8[a]	
				15% RH	2.9[a]	
				37% RH	3.2	
				62% RH	3.2[a]	
				100% RH	3.2[a]	
Veneman et al.[21]	Lewatit VP OC 1065	298	40 Pa	dry	1.1[a]	Breakthrough
				15% RH	1.3[a]	
				50% RH	1.4[a]	
		308		dry	0.9[a]	
				23% RH	1.1[a]	
				27% RH	1.2[a]	
				60% RH	1.3[a]	
Young et al.[1]	Lewatit VP OC 1065	298	400 ppm	dry	0.9[a]	Gravimetric
				30% RH	1.4[a]	
				55% RH	1.5[a]	

Wang and Li[22]	HP20 (resin), PEI	298	330 ppm	dry	1.25[a]	Breakthrough
				30% RH	1.35[a]	
				50% RH	1.35[a]	
				60% RH	1.35[a]	
				70% RH	1.35[a]	
Wang et al.[23]	SBA-15, PEI	323	10 vol.%	dry	0.85[a]	Breakthrough
				2.5 kPa	0.85[a]	
				6 kPa	1.17[a]	
				10 kPa	1.2[a]	

[a] Values estimated from figures.

References

(1) Young, J.; García-Díez, E.; Garcia, S.; Van Der Spek, M. The impact of binary water-CO_2 isotherm models on the optimal performance of sorbent-based direct air capture processes. *Energy Environ. Sci.* **2021**, *14*, 5377–5394.

(2) Ruthven, D. M. *Principles of adsorption and adsorption processes*; John Wiley & Sons, 1984.

(3) Kärger, J.; Ruthven, D. M.; Theodorou, D. N.; others *Diffusion in nanoporous materials*; Wiley Online Library, 2012; Vol. 48.

(4) Stampi-Bombelli, V.; Storione, A.; Grossmann, Q.; Mazzotti, M. On Comparing Packed Beds and Monoliths for CO2 Capture from Air Through Experiments, Theory, and Modeling. *Ind. Eng. Chem. Res.* **2024**, *63*, 11637–11653.

(5) Hahn, M. W.; Steib, M.; Jentys, A.; Lercher, J. A. Mechanism and kinetics of CO_2 adsorption on surface bonded amines. *J. Phys. Chem. C* **2015**, *119*, 4126–4135.

(6) Bollini, P.; Brunelli, N. A.; Didas, S. A.; Jones, C. W. Dynamics of CO_2 adsorption on amine adsorbents. 2. Insights into adsorbent design. *Ind. Eng. Chem. Res.* **2012**, *51*, 15153–15162.

(7) Kalyanaraman, J.; Fan, Y.; Lively, R. P.; Koros, W. J.; Jones, C. W.; Realff, M. J.; Kawajiri, Y. Modeling and experimental validation of carbon dioxide sorption on hollow fibers loaded with silica-supported poly(ethylenimine). *Chem. Eng. J.* **2015**, *259*, 737–751.

(8) Li, K.; Kress, J. D.; Mebane, D. S. The mechanism of CO_2 adsorption under dry and humid conditions in mesoporous silica-supported amine sorbents. *J. Phys. Chem. C* **2016**, *120*, 23683–23691.

(9) Mebane, D. S.; Kress, J. D.; Storlie, C. B.; Fauth, D. J.; Gray, M. L.; Li, K. Transport, zwitterions, and the role of water for CO_2 adsorption in mesoporous silica-supported amine sorbents. *J. Phys. Chem. C* **2013**, *117*, 26617–26627.

(10) Avellaneda, G. L.; Denoyel, R.; Beurroies, I. CO_2/H_2O adsorption and co-adsorption on functionalized and modified mesoporous silicas. *Microporous Mesoporous Mater.* **2024**, *363*, 112801.

(11) Elfving, J.; Bajamundi, C.; Kauppinen, J.; Sainio, T. Modelling of equilibrium working capacity of PSA, TSA and TVSA processes for CO_2 adsorption under direct air capture conditions. *J. CO2 Util.* **2017**, *22*, 270–277.

(12) Elfving, J.; Sainio, T. Kinetic approach to modelling CO_2 adsorption from humid air using amine-functionalized resin: Equilibrium isotherms and column dynamics. *Chem. Eng. Sci.* **2021**, *246*, 116885.

(13) Didas, S. A.; Sakwa-Novak, M. A.; Foo, G. S.; Sievers, C.; Jones, C. W. Effect of amine surface coverage on the co-adsorption of CO_2 and water: spectral deconvolution of adsorbed species. *J. Phys. Chem. Lett.* **2014**, *5*, 4194–4200.

(14) Belmabkhout, Y.; Serna-Guerrero, R.; Sayari, A. Adsorption of CO_2containing gas mixtures over amine-bearing pore-expanded MCM-41 silica: application for gas purification. *Ind. Eng. Chem. Res.* **2010**, *49*, 359–365.

(15) Gebald, C.; Wurzbacher, J. A.; Borgschulte, A.; Zimmermann, T.; Steinfeld, A. Single-component and binary CO_2 and H_2O adsorption of amine-functionalized cellulose. *Environ. Sci. Technol.* **2014**, *48*, 2497–2504.

(16) Wurzbacher, J. A.; Gebald, C.; Piatkowski, N.; Steinfeld, A. Concurrent separation of CO_2 and H_2O from air by a temperature-vacuum swing adsorption/desorption cycle. *Environ. Sci. Technol.* **2012**, *46*, 9191–9198.

(17) He, L.; Fan, M.; Dutcher, B.; Cui, S.; dong Shen, X.; Kong, Y.; Russell, A. G.; McCurdy, P. Dynamic separation of ultradilute CO_2 with a nanoporous amine-based sorbent. *Chem. Eng. J.* **2012**, *189-190*, 13–23.

(18) Holmes, H. E.; Ghosh, S.; Li, C.; Kalyanaraman, J.; Realff, M. J.; Weston, S. C.; Lively, R. P. Optimum relative humidity enhances CO_2 uptake in diamine-appended M2 (dobpdc). *Chem. Eng. J.* **2023**, *477*, 147119.

(19) Jung, W.; Lee, K. S. Isotherm and kinetics modeling of simultaneous CO_2 and H_2O adsorption on an amine-functionalized solid sorbent. *J. Nat. Gas Sci. Eng.* **2020**, *84*, 103489.

(20) Liu, Y.; Ye, Q.; Shen, M.; Shi, J.; Chen, J.; Pan, H.; Shi, Y. Carbon dioxide capture by functionalized solid amine sorbents with simulated flue gas conditions. *Environ. Sci. Technol.* **2011**, *45*, 5710–5716.

(21) Veneman, R.; Frigka, N.; Zhao, W.; Li, Z.; Kersten, S.; Brilman, W. Adsorption of H_2O and CO_2 on supported amine sorbents. *Int. J. Greenhouse Gas Control* **2015**, *41*, 268–275.

(22) Wang, Y.; Li, G. K. The impact of co-adsorbed water on energy consumption and CO_2 productivity in direct air capture systems. *Sep. Purif. Technol.* **2025**, *354*, 129415.

(23) Wang, Y.; Chen, C.; Li, G. K. Binary Adsorption of Carbon Dioxide and Water Vapor on Amine-Impregnated Silica. *Ind. Eng. Chem. Res.* **2024**, *63*, 11559–11569.
